# Exploring the nexus between university sustainability practices and academic performance: An empirical analysis of the QS sustainability ranking and four world university rankings

**DOI:** 10.1371/journal.pone.0306286

**Published:** 2024-10-31

**Authors:** Ruth Wanjiru Irungu, Zhimin Liu

**Affiliations:** 1 College of Public Administration, Nanjing Agricultural University, Nanjing, China; 2 Institute of Higher Education, Nanjing Agricultural University, Nanjing, China; University College Dublin, IRELAND

## Abstract

Universities, as agents of change, are expected to contribute to society’s most pressing challenges, particularly the 21^st^ century’s central issue of sustainability. Amid growing expectations from governments, society, and an increasingly conscientious student body, universities have undergone significant institutional adjustments to incorporate sustainability into their core missions of education, research, and outreach. As universities worldwide increasingly engage in sustainability practices, the question arises: How do these sustainability endeavours correlate with academic performance on a global scale? This article, using data from the QS Sustainability Ranking and four prominent academic ranking (THEWUR, ARWU, QSWUR and USWUR), investigates this link. The study explores whether sustainability relates to the academic performance of universities, the validity of the relationship when academic scores of the four rankings are aggregated, and its dependence on country-level sustainability performance scores. Findings reveal that sustainability practices have a reflection on the university rankings, providing a global competitive advantage for universities. While this study incorporates aggregated scores as a methodological innovation addressing the lack of uniformity among ranking systems, it recommends the inclusion of university-level control variables (such as faculty expertise, university budget, infrastructure) and government and policy variables in future studies to ensure robustness of the results.

## Introduction

Since its ascension to international platforms through the Brundtland Commission Report, the concept of sustainability based on three pillars (Economic, Social, and Environment) has become the most prominent socio-political agenda at the global level [1]. Subsequently, the formal recognition of higher education for sustainability occurred during the 1972 United Nations Conference on Human Environment, marking the integration of sustainability principles into academia. Some years later, in the wake of the Agenda 21 and the Rio Declaration on Environmental and Development (1992), which called for collaboration across key societal sectors, the United Nations declared the period from 2005 to 2014 as the Decade for Education for Sustainable Development. This mandate established an international mandate to incorporate sustainability principles, values, and practices into all aspects of education processes. In higher education institutions, this mandate required the integration of sustainability into education, research, operation and evaluation [2]. In response, worldwide higher education institutions have begun to adapt their missions and current practices to incorporate sustainability and have committed to various international agreements and conventions such as the Talloires Declaration, the Bologna Charter, the Halifax Declaration, and the Copernicus Charter for Sustainable Development.

The increasing attention to sustainability in higher education institutions has given rise to a research stream that delves into the intricacies of sustainable practices within universities, aiming to explore, understand, and enhance their ecological and social impact. These studies reveal that universities, as active change agents in the pursues of sustainable development, have undergone series of adjustments in order to include sustainability in their activities [3]. As universities worldwide increasingly acknowledge their role in fostering sustainability, the question arises: How do these sustainability endeavours correlate with academic performance on a global scale?

This empirical study endeavours to address this critical intersection by examining the relationship between university sustainability practices and academic performance. The primary focus of this study centres on utilizing a comprehensive dataset from different sources. By doing so, this research identifies which dimensions of sustainability have a reflection on academic performance of universities. Additionally, the influence of country-level sustainability practices on the relationship is investigated. Utilizing the university rankings from four renowned academic ranking systems, namely *Times Higher Education World University Ranking (THEWUR)*, *Academic Ranking of World Universities (ARWU)*, *The Quacquarelli-Symonds (QS) World University Ranking (QSWUR)*, *The US & World Report Best Global Universities Ranking (USWUR)*, this study empirically investigates whether sustainability practices of the universities which are ranked by the *QS Sustainability Ranking (QSSR)* explain their academic performance. The QSSR is a comprehensive assessment that measures universities worldwide based on their commitment and performance in promoting sustainability. Introduced in 2022, the QSSR incorporates indicators designed to measure an institution’s ability to tackle the world’s greatest environmental and social challenges. This research presents the first attempt to relate QSSR rankings to the academic rankings, and the findings are expected to fill the gap in the literature by providing valuable insights on promoting sustainability practices of the universities.

The rationale for utilizing the QSSR in this study is underpinned by a careful consideration of its distinct merits. Firstly, the QSSR comprises of relatively large sample (over 700 universities from 71 countries). Secondly, in comparison with alternative sustainability ranking systems, namely the UI Green Metric and Times Higher Education Impact Ranking, the QSSR offers a more comprehensive assessment of university sustainability practices, encompassing both environmental and social impact. This is unlike the UI Green Metric which primarily focuses on environmental aspects. Thirdly, while the UI Green Metric’s information sources are unclear, and only bibliometric information is evident for the THE-IR [4], the QSSR utilizes stands out by utilizing a robust methodology that combines self-reported data from universities with third-party information, enhancing reliability and credibility in its assessment process. This, coupled with the fact that the QSSR is a relatively new ranking system with no prior literature focusing on it, positions it as an intriguing case study within the scope of this research.

## Literature review

### Assessment and measurement of university sustainability strategies

In the recent past, there has been an increase in research focusing on the assessment and measurement of university sustainability practices. This has come about because of the increasing recognition of the importance and benefits of universities reporting their sustainability practices. According to Hansen, Stiling [5], the participation of a university in SDG reporting provides benefits such as discussions among units and offices about sustainability that otherwise may not have taken place. Several other studies discuss the importance of assessment and reporting to success in SDG strategies of universities. A worldwide survey that is applied to universities spanning 17 countries by Leal Filho, Shiel [6] recommends that universities should not only align their strategies towards SDGs but also integrate formal measures and reporting. Inspired by this study, Kioupi and Voulvoulis [7] develop an assessment tool for universities to evaluate their SDG practices. The tool is based on a systemic grouping of the SDGs into eight sustainability attributes, which was applied to compare 40 sustainability programmes of UK and European universities, concluding that the tool can generate empirical evidence on the effectiveness of university programmes.

Although Kioupi and Voulvoulis (2020)’s findings provide valuable insights, it is a not a straightforward task to fit the measurement of sustainability practices to a universal standardization. However, there have been recent attempts to assess sustainability practices at universities. For instance, Sánchez-Carracedo, Segalas [8] propose three tools to assess sustainability in university education, specifically engineering. Similarly, Cottafava, Ascione [9] propose a novel multi-step methodology for assessing sustainability-related research at universities and applies the tool at an Italian generalist university (University of Turin). De la Poza, Merello [10] attempts to assess the level of SDG knowledge among university students in Spain. Using big data technology [11], examine the visibility of information about the SDGs on the websites of Spanish and major international universities. In the US [5], assess the efforts of University of South Florida in integrating the SDGs.

Beyond the focus on sustainability initiatives centred on the primary missions of education and research, scholars have also endeavoured to assess the role of university outreach, the third mission, in advancing sustainability. Outreach on sustainability, defined as a universities’ actions to engage with the communities in its surroundings in mutually beneficial process of sustainable development, is highlighted by Berchin, de Aguiar Dutra [12] as a potential key contributor to sustainability. Some researchers have even argued that it is through outreach that universities might be able to contribute to sustainability the most, through connecting to the academic and local communities [13].

In terms of evaluation tools for university outreach on sustainability, several tools have been designed. For instance, Plummer, Witkowski [14] developeds a tool, the HEI-Community Partnership Performance Index (HCPPI). The tool was used to examine three HEI-community partnerships for sustainability science in Canada (the Brock-Lincoln Living Lab, the Excellence in Environmental Stewardship Initiative, and Niagara Adapts). The results showed it is important to incorporate systemic performance assessments into HEI-community partnerships, as it can promote accountability, transparency, and continuous improvement. This is important in sustainability science, which requires constant reflection, adaptation, and learning.

In another study [15], the paradigm of sustainability within the third mission practices of universities has undergone a conceptual redefinition, now characterized as “co-creation for sustainability”, and denoted as an emerging, new mission for the university. The study argues that this emerging mission distinctively departs from the traditional economic focus of the third mission and conventional technology transfer practices. To fulfil this emerging mission, the study identifies five channels that universities can utilize, namely knowledge management, technical demonstration projects and experiments, technology transfer and economic development, reform of built and natural environment, and socio-technical experiments.

By utilizing data from the United Nations Principles of Responsible Management Education (UNPRME), Avelar, da Silva Oliveira [16] investigated how HEIs are integrating the SDGs into their curricula, research, and partnerships. The UNPRME is the largest voluntary engagement platform for academic institutions to transform their teaching, research, and thought leadership in support of universal values of sustainability, responsibility, and ethics. Their findings revealed that curricula in HEIs has been modified to include new courses, modules, and disciplines that address the SDGs, while research and partnerships demonstrate cooperative behaviour between HEIs, companies, society and governmental and non-governmental organizations institutions [16].

In addition to examining university sustainability practices, researchers have turned their attention to ranking systems specifically designed to assess these practices. These ranking systems include the Times Higher Education Impact Ranking (THE-IR), which emerged in 2019, and assesses universities’ impacts against the SDGs as well as the UI Green Metric that was introduced by the University of Indonesia in 2010 and ranks universities for their activities related to green campus and sustainability. The rankings have increasingly gained recognition in academic literature as the two prominent sustainability rankings, as highlighted by Torabian [17]. For instance, Bautista-Puig, Orduña-Malea [18] analysed the methodology, data, and coverage of THE-IR. Their study revealed severe inconsistencies in the THE-IR methodology, and they argued that the ranking system offers a distorted view of sustainability in universities. The methodological issues with the THE-IR were also noted by another study [4], which evaluated both the THE-IR and UI Green Metric based on the Berlin Principles framework. Still, it was noted that the THE-IR had a better performance than the UI Green Metric, signifying the need for future research and development in sustainability rankings to address and rectify the identified inconsistencies to ensure the reliability and accuracy of such evaluation systems. While the QS has introduced a new sustainability ranking, the QSSR, there are still no studies on this new ranking system. This absence of research positions the QSSR as an intriguing case study within the scope of this research, providing an opportunity to explore and analyse its methodologies, impact, and potential contributions to the assessment of sustainability practices in universities.

### Impacts of sustainability practices on universities

Enhanced institutional reputation has been identified as one of the perceived benefits of embracing sustainability practices within a university. As societal awareness and concern for environmental issues grow, universities that actively demonstrate a commitment to sustainability are viewed favourably by stakeholders and the broader community. Such institutions are often perceived as socially responsible and forward-thinking, attracting individuals who value environmental stewardship and ethical considerations in their academic pursuits. In their study, Sassen, Azizi [19] investigated the motivations for disclosing sustainability information by US universities. The study revealed that the establishment and disclosure of sustainability practices within a university are predominantly motivated by a desire to meet societal expectations. Furthermore, an enhanced institutional reputation is heavily tied with other benefits such as attracting prospective students.

Attracting more students, particularly among private universities that heavily depend on tuition fees is a motivating factor for universities to engage in sustainability practices. This motivation aligns with the growing recognition that students, as key stakeholders, are increasingly demanding that their prospective or current universities be more environmentally sustainable [20]. In survey of more than 2,000 prospective international students by Times Higher Education (THE), the vast majority (79%) revealed that they believe that universities have an important role to play in advancing the SDGs. Furthermore, the prospective students were more likely to choose a university based on its commitment to sustainability than for its location. Similarly, in a 2022 survey by Quacquarelli-Symmonds (QS) on over 3,000 prospective international students, the students identified social sustainability as a determining factor when choosing a university. Among them, 71% selected ‘human rights’ as one of the values they would like to see in a university they study at, followed by ‘commitments to ethical working practices’ (64%) [21]. These sentiments are echoed by a recent study [22], that a university deemed sustainable by students becomes more attractive to them. As such, this connection between sustainability initiatives and student attraction underscores the pragmatic and strategic considerations that universities weigh in establishing sustainability practices.

The interaction between universities and sustainability also brings benefits such as new funding flows [23]. In US, financial advantages associated with sustainability were also noted by Sassen, Azizi [19] to be a motivating factor for sustainability practices among universities. Their study revealed that attracting stakeholder donations through the fulfilment of societal expectations was the most significant motivation of sustainability practices and disclosures for U.S. universities. The study recommends that small universities could disclose sustainability-related information on a voluntary basis to achieve a unique selling point and increase their competitiveness [19].

Furthermore, sustainability practices in higher education have been associated with improved academic performance. Sustainability often includes experiential learning, a learning approach that takes place beyond the traditional classroom of teaching about sustainability and into fostering a change through teaching and learning for and as sustainability [24], through projects such as internships, living lab projects, and other hands-on experiences [25]. These experiences have been proven to aid students’ academic achievements and graduate attributes [26]. In a study that explored the discourse on experiential approaches to higher education for sustainability, Backman, Pitt [27] revealed that experiential learning promotes desirable learning outcomes and skills development among students.

## Data and methodology

To empirically investigate the relationship between university sustainability practices and academic performance, a comprehensive data collection protocol encompassing four distinct university ranking systems was implemented. Notably, the QSSR Ranking served as the primary instrument for gauging the efficacy of universities in sustainability practices. Concurrently, academic performance metrics were sourced from four renowned university systems, namely (ARWU), QSWUR, USWUR, and (THEWUR). Each ranking system offers unique insights into university performance: THEWUR highlights teaching, research, citations, international outlook, and industry income; ARWU emphasizes Nobel laureates, highly cited researchers, and prestigious publications; QSWUR prioritizes academic and employer reputation, faculty-student ratio, and citations; while USWUR focuses on academic research and reputation. This diversity underscores the multifaceted nature of evaluating global universities, facilitating a comprehensive examination of sustainability practices and academic excellence.

The sample includes all universities which have both a QSSR and at least one academic performance score provided by the three ranking systems in the year 2023. Table 1 summarizes the number of universities included in each of the four ranking systems with the number of factors considered in their methodologies. This methodologically rigorous approach ensures a nuanced examination of the intricate interplay between sustainability initiatives and academic achievements, leveraging multiple authoritative sources for a robust analysis within the higher education domain. Table 1 summarizes the number of universities included in each of the four ranking systems with the number of factors considered in their methodologies.

**Table 1:** The four ranking systems and number of universities included.

Ranking system	Number of universities	Number of factors
QSSR Ranking	700	8
THEWUR	651	5
QSWUR	417	6
USWUR	682	13
ARWU	92	6

### QSSR Ranking (QSSR)

The QSSR is a comprehensive assessment that evaluates universities worldwide based on their commitment and performance in promoting sustainability. The QSSR was first issued in 2022 and comprised of 700 universities from 71 different countries. The QSSR comprises of indicators designed to measure an institution’s ability to tackle the world’s greatest environmental and social challenges. Indicators of the QSSR are split into two: First is environmental sustainability measures, which includes *sustainable institutions; sustainable education*, *and sustainable research*. Second is social impact measures, which include *equality*, *knowledge exchange*, *educational impact*, *employability and opportunities*, *and quality of life*. The weights of each of these sub-dimensions and the indicators used within the scope of each sub-dimension are provided in Table 2.

**Table 2:** QSSR Ranking sub-dimensions and their corresponding weights.

Factors	Weights	Indicators
Sustainable Institutions	17.5%	Alumni impact for innovation, member of an officially recognised sustainable group, climate change commitment (staff view), publicly available strategy on sustainable procurement, publicly available strategy on sustainable investment, student society focused on environmental sustainability, report on emissions/ energy/ water use, Race to Zero commitment.
Sustainable Education	20%	Academic reputation for sustainable education, alumni impact for environmental sustainability, research centre focused of environmental sustainability, climate science and/or sustainability courses
Sustainable Research	12.5%	Research impact on environment-aligned SDGs, national statistics for research, research centre with sustainability focus, policy citations (environmental)
Equality	15%	Research impact into SDGs for equality; student gender ratio; faculty gender ratio; women in leadership ratio; equality, diversity, and inclusion policy; academic equality; office for disability; equality national statistics.
Knowledge Exchange	10%	Knowledge exchange: progress/dissemination; research partnerships with employers.
Impact of Education	10%	Research impact into SDGs for education; academic reputation for impact of education; alumni impact for education; academic freedom index; impact of education national statistics
Employability and Opportunities	10%	Employer reputation; employment outcomes; research impact into SDGs for employment and opportunities; job preparedness (graduates view); employment and opportunities national statistics
Quality of Life	5%	Research impact for SDGs for quality of life; health options on campus; quality of life national statistics

**Fig 1:**
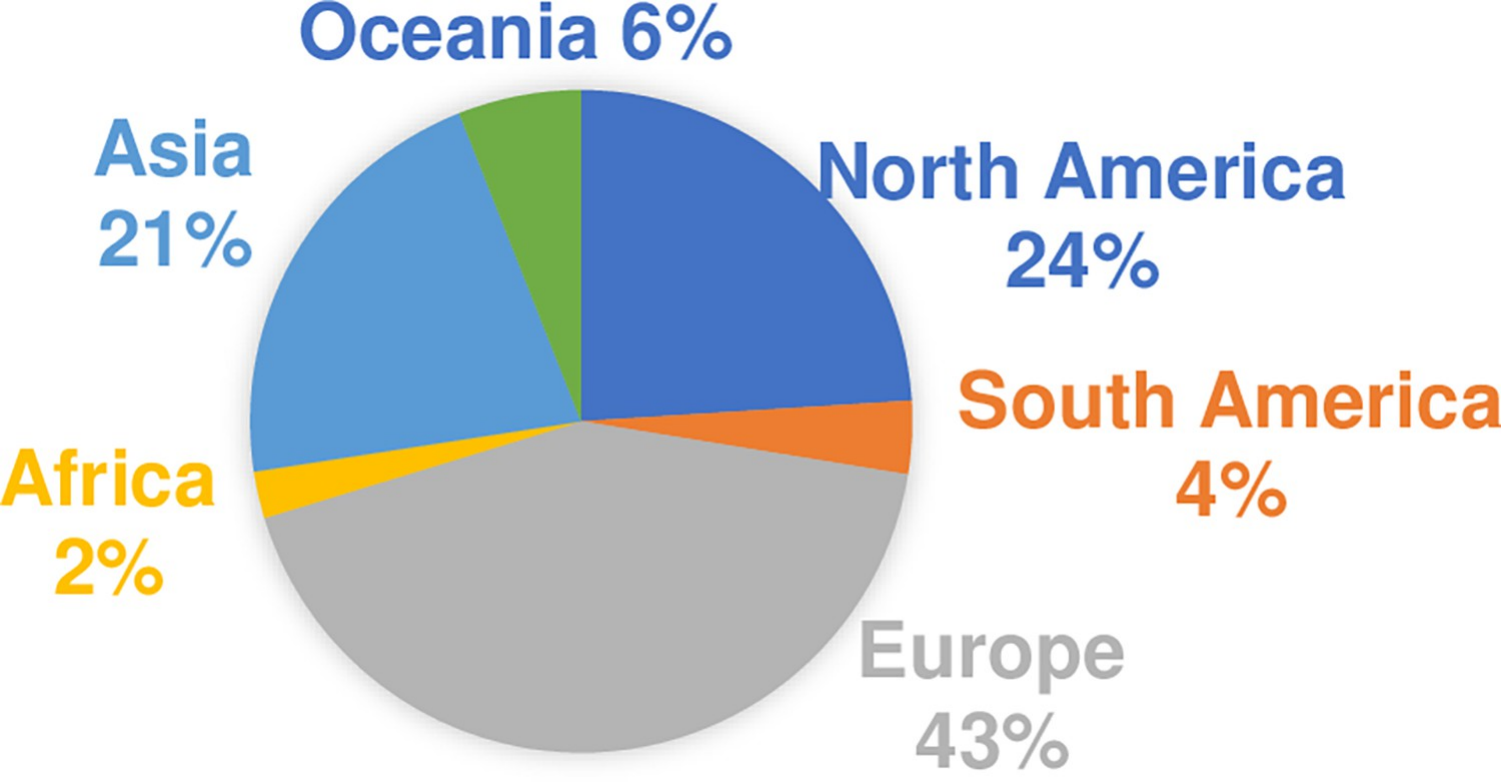
The shares of different world regions in the QSSR (2023).

Fig 1 shows the shares among different world regions in the QSSR. Europe takes the lead with 43%, followed by North America (24%) and Asia (21%) while Africa is the least represented (2%). Furthermore, The United States of America leads with 136 universities (19.4%), followed by the United Kingdom with 68 universities (9.7%), Germany with 40 universities (5.7%), China with 37 universities (5.2%), and Australia with 34 universities (4.8%).

### Times Higher Education World University Ranking (THEWUR)

THEWUR is an annual publication of global university rankings that was initiated in 2004. THEWUR uses 13 performance indicators grouped into 5 sub-dimensions: teaching (the learning environment); research (volume, income, and reputation); citations (research influence); international outlook (staff, students, and research); and industry income (knowledge transfer). Table 3 shows the weights of each of these sub-dimensions and the indicators used within the scope of each sub-dimension are provided.

**Table 3:** THEWUR sub-dimensions and their corresponding weights.

	Factors	Weights (%)
**1**	Teaching (the learning environment)	30
**2**	Research (volume, income, and reputation)	30
**3**	Citations (research influence)	30
**4**	International outlook (staff, students, research)	7.5
**5**	Industry income (knowledge transfer)	2.5

### Academic Ranking of World Universities (ARWU)

ARWU is an academic ranking system of global universities that was initiated by the Centre for World-Class Universities at the Institute of Higher Education of Shanghai Jiao Tong University in 2003. Since 2009, the ARWU has been published and copyrighted by an independent organisation (Shanghai Ranking Consultancy). ARWU uses six objective indicators which include: number of alumni and staff winning Nobel Prizes and Field Medals; number of highly cited researchers selected by Clarivate, number of articles published in journal of Nature and Science, number of articles indexed in Science Citation Index Expanded and Social Sciences Citation Index in the Web of Science, and per capita performance of a university. The ARWU ranks more than 2500 universities each year and publishes the best 1000. Table 4 shows each of the six sub-dimensions and their corresponding weights.

**Table 4:** ARWU sub-dimensions and their corresponding weights.

	Factors	Weights (%)
1	Alumni of an institution winning Nobel Prizes and Field Medals	10
2	Staff of an institution winning Nobel Prizes and Fields Medals	20
3	Highly cited researchers	20
4	Papers published in Nature and Science	20
5	Papers indexed in Science Citation Index-Expanded and Social Science Citation Index	20
6	Per capita academic performance of an institution	10

### QS World University Ranking (QSWUR)

The QSWUR is an annual publication of global university rankings. During 2004–2009, it was pursued in cooperation with Times Higher Education, and its fundamental criteria included research, employability, teaching, and internationalization. However, since 2010, it has been published independently and until 2023, included six sub-dimensions: academic reputation; employer reputation; faculty student ratio; citations per faculty; international faculty ratio; and international student ratio. In its most recent ranking, the 2024 ranking, the QSWUR significantly evolved its methodology to include three new indicators, including international research network, employment outcomes, and sustainability, which reflects the shifts in higher education in the past two decades. This research utilizes the 2023 ranking, and Table 5 shows the six sub-dimensions along with their respective weights for the 2023 ranking.

**Table 5:** QSWUR sub-dimensions and their corresponding weights.

	Factors	Weights (%)
1	Academic reputation	40
2	Employer reputation	10
3	Faculty student ratio	20
4	Citations per faculty	20
5	International faculty ratio	5
6	International student ratio	5

### US news & World Report Best Global Universities Ranking

The USWUR has been produced annually for the past nine years to provide insight into how universities compare globally. It focuses on specifically on schools’ academic research and reputation and the 2023 ranking encompasses 2,000 top institutions from 95 countries. The USWUR uses 13 indicators to rank the institutions as shown in Table 6.

**Table 6:** USWUR sub-dimensions and their corresponding weights.

	Factors	Weights (%)
1	Global research reputation	12.5
2	Regional research reputation	12.5
3	Publications	10
4	Books	2.5
5	Conferences	2.5
6	Normalized citation impact	10
7	Total citations	7.5
8	Number of publications that are among the 10% most cited	12.5
9	Percentage of total publications that are among the 10% most cited	10
10	International collaboration—relative to country	5
11	International collaboration	5
12	Number of highly cited papers that are among the top 1% most cited in their respective field	5
13	Percentage of total publications that are among the top 1% most highly cited papers	5

### Analysing the relationship between QSSR scores and the academic performance of universities in each of the four academic ranking systems

In the initial phase of the empirical investigation, separate regressions are conducted for QSSR scores, utilizing the academic performance data from four distinct ranking systems: THEWUR; ARWU; QSWUR; and USWUR. The matching process involves identifying universities present in both the QSSR and each of the four academic ranking systems, determining the sample size for each estimation based on the specific academic ranking system being examined. The varying sizes of academic ranking systems are illustrated in Table 7. QSSR & USWUR dataset has the highest number of universities with 682, which can be attributed to the high number of universities included in the USWUR system in 2023, which comprised 2,000 global universities (the highest among the four ranking systems).

**Table 7:** The intersection of QSSR with each of the academic ranking systems.

Data sets	Number of universities in both data sets
QSSR & THEWUR	651
QSSR & ARWU	92
QSSR & QSWUR	417
QSSR & USWUR	682

To analyse the relationship between QSSR scores and the academic performance of the universities, the following model is estimated:

$${AS}_{i}=\alpha +\beta {QSSR}_{i}+{Country}_{c}+\varepsilon_{i}$$ (Eq 1)

In Eq (1), *AS*_*i*_ corresponds to the scores of a university in the four different academic rankings systems, namely THEWUR; ARWU; QSWUR; and USWUR in 2023. *QSSR*_*i*_ represents eight different QSSR Ranking sub-scores, namely *sustainable institutions sustainable education*, *sustainable research*, *equality*, *knowledge exchange*, *educational impact*, *employability and opportunities*, *and quality of life* as well as the two overall QSSR categories: *Environmental Impact and Social Impact* of the university in 2023. *β* is the coefficient of QSSR score, *α* is the constant term, and *ε* denotes the error terms of the estimation.

Consideration of the characteristics of the country in which a country is situated is a crucial factor in our analysis. It is reasonable to anticipate variations in financial strength in terms of GDP per capita, as well as disparities in educational history and research funding across different countries. Notably, certain countries, such as the USA dominates the top-ranking positions in almost all the different global university ranking systems, closely followed by the UK and China. To mitigate the risk of omitted variable bias, it is important to incorporate country-specific factors into our estimation model. To control for unobserved country-specific effects, we include country dummy variables (denoted as *Country*_*c*)_ in all our estimations. These dummy variables account for inherent differences between countries that may influence the performance of universities. Furthermore, to address the potential issue of variance heterogeneity, we employ heteroscedasticity-robust standard errors in our estimations. This robust approach ensures that our model accounts for variations in variance across different observations, enhancing the reliability of our results.

## Results and discussions

### The relationship between QSSR scores and the academic performance of universities in each of the four academic ranking systems

Table 8 presents the results regarding the relationship between QSSR Ranking (QSSR) and academic performance scores provided by four different academic ranking systems. Demonstrating positive coefficients, both the QS Environmental Impact and Social Impact provide significance across all 4 academic ranking systems (THEWUR, QSWUR, USWUR and ARWU) in explaining the academic performances. These results indicate that a substantial proportion of the variance in university academic performances is accounted for by the sustainability policies and performance metrics of the universities, as quantified by the QSSR. Furthermore, the positive coefficients associated with the QSSR Environmental and Social Impact scores indicate a positive relationship between sustainability performance and academic performance. Specifically, universities with higher sustainability scores exhibit higher academic performance scores. This observation holds across all academic ranking systems, implying a consistent association.

**Table 8:** Relationship between academic score and QSSR total scores.

	THEWUR	QSWUR	USWUR	ARWU
**QS Environmental Impact Total Score**	0.000***	0.006**	0.000***	0.000***
	(0.081)	(0.098)	(0.064)	(0.071)
Constant	0.000***	0.000***	0.000***	0.026*
	(3.911)	(2.674)	(4.472)	(4.470)
R^2^	0.493	0.127	0.487	0.267
**QS Social Impact Total Score**	0.000***	0.000***	0.000***	0.017*
	(0.019)	(0.062)	(0.013)	(0.132)
Constant	0.000***	0.000***	0.000***	0.887
	(1.729)	(2.485)	(2.948)	(11.34)
R^2^	0.697	0.272	0.774	0.209
# of observations	651	417	682	92

Notes: country-fixed effects are included in all estimations. Robust standard errors are in parenthesis.

*P<0.05

** P<0.01

*** P<0.001

The implications of these findings underscore the potential interplay between sustainable practices and academic excellence in higher education institutions. The positive correlation suggests that universities prioritizing sustainability initiatives may not only contribute to environmental and social well-being but also enhance their academic standing. This aligns with the growing recognition of the interconnectedness between institutional sustainability efforts and overall institutional performance as well as students’ academic achievements and graduate attributes [26, 27].

As a subsequent investigation, the nexus between the academic performance of universities and the 8 sub-dimensions of the QSSR (*sustainable institutions sustainable education*, *sustainable research*, *equality*, *knowledge exchange*, *educational impact*, *employability and opportunities*, *and quality of life*) are tested. This analytical step allows us to identify which aspects of university sustainability practices, as measured by the QSSR, wield notable influence over academic performance. Such nuanced insights can inform targeted strategies for universities aiming to bolster both their sustainability and initiatives and academic standing. The results are outlined in Table 9.

**Table 9:** Relationship between academic scores and QSSR factors.

	THEWUR	QSWUR	USWUR	ARWU
***Panel A*. *Sustainable Institutions (SI)***				
*SI*	0.000***	0.001**	0.001**	0.004**
	(0.029)	(0.060)	(0.024)	(0.056)
Constant	0.000***	0.000***	0.001**	0.000***
	(2.166)	(0.852)	(5.261)	(3.183)
R^2^	0.398	0.080	0.324	0.167
***Panel B*. *Sustainable Education (SE)***				
*SE*	0.019*	0.116	0.008**	0.822
	(0.044)	(0.075)	(0.033)	(0.030)
Constant	0.000***	0.000***	0.000***	0.000***
	(2.841)	(4.000)	(4.034)	(2.077)
R^2^	0.372	0.079	0.309	0.095
***Panel C*. *Sustainable Research (SR)***				
*SR*	0.000***	0.000***	0.000***	0.001**
	(0.019)	(0.041)	(0.013)	(0.061)
Constant	0.000***	0.000***	0.000***	0.004**
	(1.804)	(0.868)	(2.850)	(4.738)
R^2^	0.541	0.205	0.609	0.177
***Panel D*. *Equality (EQ)***				
*EQ*	0.000***	0.003**	0.000***	0.917
	(0.033)	(0.070)	(0.024)	(0.072)
Constant	0.000***	0.000***	0.000***	0.000***
	(2.905)	(1.040)	(7.123)	(5.704)
R^2^	0.440	0.077	0.426	0.095
***Panel E*. *Knowledge Exchange (KE)***				
*KE*	0.000***	0.000***	0.000***	0.005***
	(0.055)	(0.049)	(0.012)	(0.109)
Constant	0.000***	0.000***	0.000***	0.855
	(3.289)	(3.378)	(1.754)	(9.684)
R^2^	0.537	0.224	0.706	0.217
***Panel F*. *Impact of Education (IE)***				
*IE*	0.000***	0.000***	0.000***	0.021*
	(0.045)	(0.063)	(0.044)	(0.106)
Constant	0.000***	0.000***	0.000***	0.374
	(3.367)	(3.149)	(3.920)	(9.166)
R^2^	0.552	0.162	0.553	0.190
***Panel G*. *Employability and Opportunities (EO)***				
*EO*	0.000***	0.000***	0.000***	0.000***
	(0.018)	(0.051)	(0.0142)	(0.069)
Constant	0.000***	0.000***	0.000***	0.831
	(1.593)	(3.139)	(2.759)	(6.389)
R^2^	0.728	0.396	0.697	0.301
***Panel I*. *Life Quality (LQ)***				
*LQ*	0.000***	0.000***	0.000***	0.231
	(0.017)	(0.047)	(0.011)	(0.162)
Constant	0.000***	0.000***	0.000***	0.464
	(1.776)	(1.916)	(1.986)	(15.023)
R^2^	0.6177	0.184	0.708	0.122
# of observations	650	417	682	92

Notes: country-fixed effects are included in all estimations. Robust standard errors are in parenthesis.

*P<0.05

** P<0.01

*** P<0.001

The results in Table 9 provide several interesting insights. First, 5 of the 8 sub-dimensions of the QSSR Ranking (*Sustainable Institutions*, *Sustainable Research*, *Knowledge Exchange*, *Impact of Education*, *and Employability & Opportunities*) hold a strong positive relationship with the academic scores of all four academic ranking systems. On the other hand, *Equality (EQ) and Life Quality (LQ)* show significance with only three of the academic ranking systems (THEWUR, QSWUR, and USWUR). However, *Sustainable Education (ES)* only has a significant relationship with the academic ranking scores of THEWUR and USWUR.

From the perspective of the academic ranking systems, the results show that most of the QSSR dimensions provide significance in explaining the THEWUR and USWUR, followed by QSWUR, and last is ARWU which shows significance with only 5 of the 8 sub-dimensions. One plausible explanation for this pattern is the comparatively smaller number of universities (92) included in the ARWU. This reduced sample size may impact the statistical power of the analysis, potentially leading to fewer significant associations. In contrast, the larger datasets of THEWUR (650) and USWUR (682) offer a more robust basis for detecting significant relationships with a broader range of QSSR dimensions.

### The relationship between QSSR scores and aggregated academic performance

While investigating the relationship between the QSSR and academic performance scores across the 4 academic ranking systems yields significant insights, we suggest that using an aggregate academic performance may enhance reliability and stability. The rationale to use an aggregated academic performance score is underpinned by various considerations. First is the absence of uniformity among global university ranking systems, which is marked by a diversity of criteria and methodologies. This diversity leads to a dearth of agreement concerning the definitive measure of academic excellence. Notably, a majority of the ranking systems do not have a definition of quality that is clear and based on a theory, and as such, it can be stated that there is little agreement on what demonstrates quality [28]. This disagreement is evident in the results of the rankings, where there is a large variation among the top one hundred universities between different rankings. As such, using an aggregated academic score helps mitigate these methodological differences among ranking systems, providing a more comprehensive and balanced representation of academic performance. Secondly, the outcomes presented in Table 9 demonstrate a significant sensitivity to the choice of academic ranking system. For instance, various academic ranking systems, such as THEWUR and USWUR show significance with more of the QSSR subdimensions than ARWU and QSWUR. Thirdly, using an aggregation scheme of the four academic ranking systems serves as a robustness check by increasing the sample size to 694 universities with a sustainability score and a composite academic performance score.

According to [29], while creating such an aggregation scheme, several factors should be considered. First is that any university being excluded from a ranking system does not necessarily indicate a poor performance. This is because different rankings represent different views on quality in higher education, leading to the exclusion of certain universities from specific ranking systems [28]. As such, it would be misleading to add total scores to end up with a composite score, as this would lead to a dramatic negative effect on the score of a university that is not included in a ranking system. Second is avoidance of the use of simple averages, where a university that is included in a limited number of lists with a high score would dominate a university included in several ranking systems with relatively low scores.

As such, the proposed Aggregated Academic Score is as follows:

$${AAS}_{i}=\left(\sum_{\begin{array}{c} i\in M\\ j\in N \end{array}}\frac{s_{ij}}{r_{ij}}\right)\times t_{i}$$ (Eq 2)

*M* represents a set of universities with at least one score in academic ranking systems

N represents a set of academic ranking systems.

*S*_*ij*_ represents the score of university *i* in a ranking system. *r*_*ij*_ represents the rank of a university in a ranking system. *t*_*i*_ represents the number of times university *i* is included in different ranking systems divided by four.

The formula in Eq 2 helps obtain a single composite academic performance score, which takes into account the total scores in each system, ranks of the universities, and also the number of times which a university is covered by the ranking systems. Then, the QSSR sub-dimensions are regressed with the AAS as follows:

$${AAS}_{i}=\alpha +\beta {QSSR}_{i}+\varepsilon_{i}$$ (Eq 3)

In Eq 3, *AAS*_*i*_ represents the composite academic performance score of university *i* and *QSSR*_*i*_ is the QSSR sub-dimensions, including the *Environmental Impact* and *Social Impact* total scores.

Table 10 shows a significant relationship between the aggregated academic score and all the sub-dimensions of the QSSR Ranking. This verifies previous results, and it can therefore be concluded that the main results are robust in differentiating considerations of academic performances.

**Table 10:** Relationship between aggregated academic score and QSSR sub-dimensions.

	ENVIRONMENTAL IMPACT	SI	SE	SR	SOCIAL IMPACT	EQ	KE	IE	EO	LQ
AAS	0.000***	0.000***	0.001*	0.000***	0.000***	0.000***	0.000***	0.000***	0.000***	0.000***
	(0.027)	(0.029)	(0.017)	(0.020)	(0.024)	(0.025)	(0.021)	(0.022)	(0.024)	(0.019)
Constant	0.004**	0.323	0.658	0.007**	0.000***	0.157	0.009**	0.000***	0.000***	0.006**
	(1.123)	(1.082)	(0.920)	(1.118)	(1.225)	(1.441)	(1.237)	(1.179)	(1.285)	(1.124)
R^2^	0.049	0.022	0.012	0.046	0.075	0.016	0.038	0.061	0.087	0.046
# of observations	694	694	694	694	694	694	694	694	694	694

Notes: The dependent variable of the regression model is the Aggregated Academic Score (AAS). country-fixed effects are included in all estimations. Robust standard errors are in parenthesis.

*P<0.05

** P<0.01

*** P<0.001

### Country-level sustainability performance

The Sustainable Development Report (SDR) is utilized in this study to offer an outlook of the sustainability performance of various countries. The SDR is an annual assessment of progress for all UN member states towards the SDGs. The 2023 ranking ranks 166 countries based on their overall score. The overall score measures the total progress towards achieving all 17 SDGs, where 100 is the highest score.

**Fig 2:**
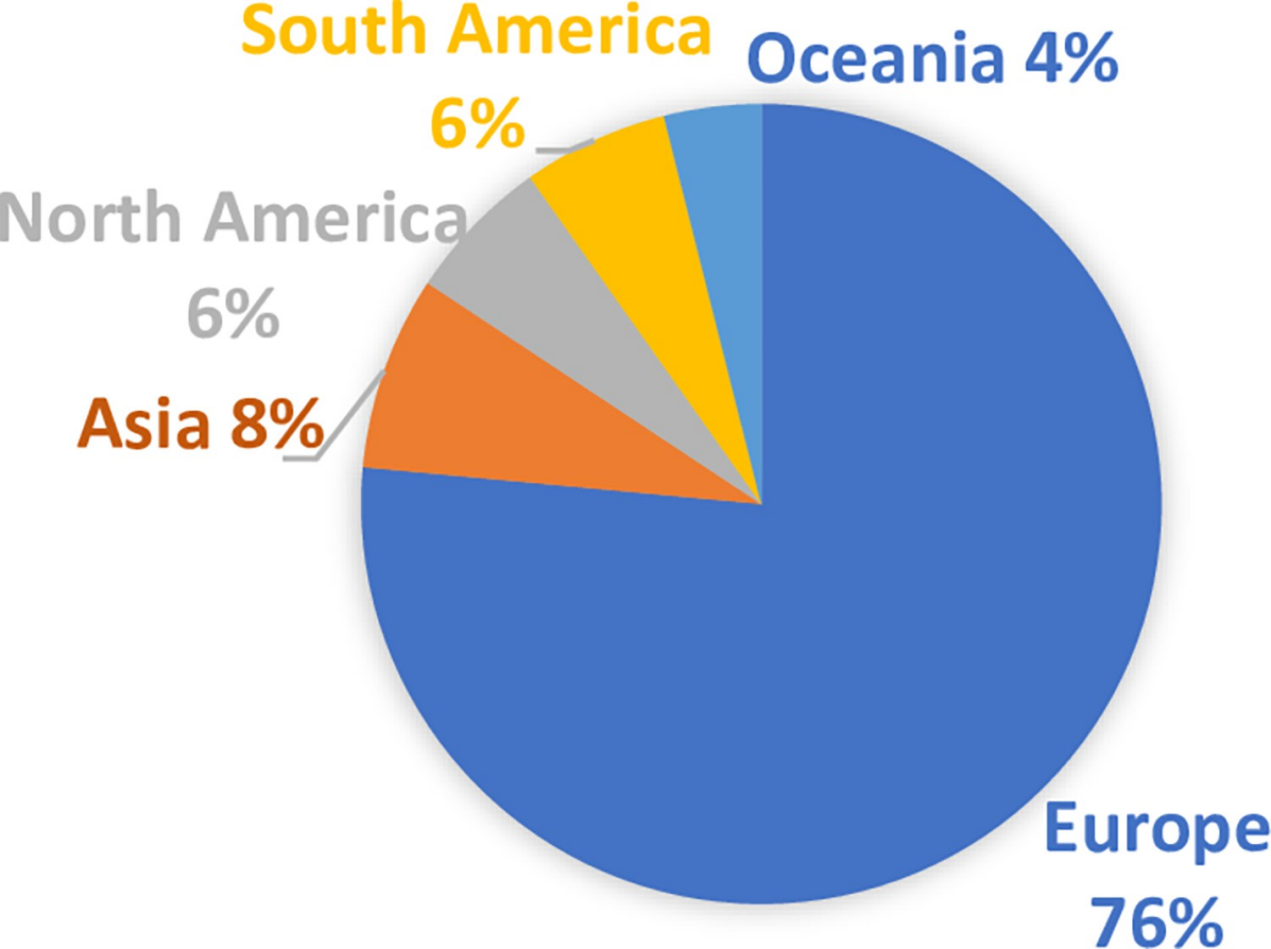
The distribution of different world regions in the Sustainable Development Report.

Fig 2 shows the distribution of various world regions within the top 50 countries ranked in the Sustainable Development Report in 2023. In addition to Europe having the largest share (76%), the first 20 countries are all from Europe, being led by Finland ((86.76), followed by Sweden (85.98) and Denmark (85.68). Other developed countries such as Japan, Canada, and USA top the ranking at positions 21, 26, and 39 respectively. This implies that universities from these countries with a superior ranking in sustainability practices could potentially possess an advantage in terms of their institutional sustainability practices.

Therefore, while country fixed effects were accounted for in all previous estimations, it does not preclude the potential moderating influence of sustainability at the country level on the relationship between QSSR scores and academic performance of universities. Given that certain countries outperform others in sustainability measures, one could posit that the relationship between QSSR scores and the academic performance of universities is more robust in nations with superior sustainability achievements. This suggests that the link between sustainability scores and academic excellence may be heightened in regions where there is a stronger commitment and overall performance in sustainable practices. The varying degrees of emphasis on sustainability across countries introduce a nuanced dimension that influences the strength of the observed relationship between sustainability scores and the academic performance of universities.

The Sustainable Development Report (SDR) is included in the model, which is as follows:

$${AAS}_{i}=\alpha +\beta_{1}{QSSR}_{i}+\beta_{2}{QSSR}_{i}{xSDR}_{j}+\varepsilon_{i}$$ (Eq 4)

In Eq 4, *SDR* represents the sustainability performance of country *j* in 2023. Other variables are previously defined. A positive coefficient of *QSSR*_*i*_*xSDR*_*j*_ (*β*_2_) indicates that higher country-level sustainability performance reinforces the relationship between sustainability scores and academic performances of the universities.

Despite the initial assumption that overall country-level sustainability performance would significantly impact the empirical analysis, the findings in Table 11 reveal a more intricate picture. The fact that only 2 (*Sustainable Education and Sustainable Research*) out of the 8 sub-dimensions from the QSSR Ranking exhibit significance when SDR country scores are included in the regression model suggests that specific aspects of sustainability, rather than the broad country-level performance, play a more influential role in moderating effects. This outcome underscores the importance of considering individual sub-dimensions within sustainability rankings, as different facets may contribute differently to the relationship between sustainability scores and academic performance at the university level.

**Table 11:** Moderating effect of country-level sustainability performance.

	ENVIRONMENTAL IMPACT	SI	SE	SR	SOCIAL IMPACT	EQ	KE	IE	EO	LQ
AAS	0.183	0.438	0.017	0.000***	0.045*	0.636	0.770	0.046	0.991	0.181
	(0.071)	(0.067)	(0.045)	(0.060)	(0.055)	(0.054)	(0.037)	(0.051)	(0.046)	(0.046)
AAS *x* SDR	0.670	0.223	0.011*	0.009**	0.442	0.963	0.257	0.329	0.104	0.751
	(0.000)	(0.000)	(0.000)	(0.060)	(0.000)	(0.000)	(0.000)	(0.000)	(0.000)	(0.000)
Constant	0.001**	0.969	0.300	0.006**	0.000***	0.385	0.115	0.000***	0.000***	0.004**
	(0.383)	(0.357)	(0.292)	(0.271)	(0.388)	(0.462)	(0.375)	(0.370)	(0.441)	(0.298)
R^2^	0.049	0.010	0.009	0.077	0.074	0.010	0.026	0.060	0.087	0.065
# of observations	694	694	694	694	694	694	694	694	694	694

Notes: This table presents the impact of country-level sustainability performance index on the relationship between the QSSR and the aggregate academic score. The dependent variable in the regression model is the Aggregate Academic Score (AAS). Robust standard errors are in parenthesis.

*P<0.05

** P<0.01

*** P<0.001

## Conclusion

This study aimed at investigating the relationship between sustainability scores and academic performance and it could generate some insights on the intricate interplay between institutional sustainability practices and the academic performance of universities. It is evident that sustainability may have a pivotal role in influencing the academic performance of universities. The consistent positive association observed across various academic ranking systems underscores the significance of sustainability as a multifaceted factor that not only contributes to environmental and social well-being but also enhances the academic standing of higher education institutions. This echoes some previous studies [19], which associate university sustainability practices with enhanced institutional reputation [19], attracting top talents (students and faculty) [20, 22], funding opportunities [23], as well as students’ academic achievements and graduate attributes [26]. This dual impact suggests a paradigm shift in viewing universities, emphasizing their potential to serve as holistic contributors to societal progress and academic excellence simultaneously.

Delving into the specific dimensions of the QSSR Ranking (QSSR) that exhibit significance with academic performance provides nuanced insights. *Sustainable Institutions*, *Sustainable Research*, *Knowledge Exchange*, *Impact of Education*, *and Employability & Opportunities* emerge as key aspects strongly correlated with academic scores. These dimensions signify the diverse facets through which sustainability practices influence academic performance. *Sustainable Institutions and Research* reflect an institution’s commitment to integrating sustainability into its core functions, fostering an environmental that prioritizes both ecological and educational values. Knowledge Exchange and Employability & Opportunities underscore the practical implications of sustainability, indicating that universities actively engaged in sharing knowledge and providing opportunities for students are more likely to achieve higher academic standing. This is in agreement with a previous study [27], which revealed that the experiential learning that is characteristic of sustainability curriculums have been proven to promote desirable learning outcomes and skills development among students.

The examination of country-level sustainability performance through the Sustainable Development Report (SDR) introduces a nuanced dimension. Specific aspects of sustainability, particularly Sustainable Education and Sustainable Research, emerge as influential moderators of effects. This underscores the need for a granular examination of sustainability metrics, acknowledging that the impact of sustainability practices on academic performance varies across regions. This insight prompts universities and policymakers to tailor strategies to the specific aspects of sustainability that hold the most significant sway within their respective contexts.

This study incorporates an aggregated academic performance score, serving as a methodological strength that mitigates the methodological differences among global university ranking systems. This approach not only enhances reliability and stability but also provides a more comprehensive and balanced representation of academic performance. The use of aggregated scores addresses the lack of uniformity among ranking systems, acknowledging the diverse criteria and methodologies utilized. This methodological innovation contributes to the study’s credibility and underscores the importance of adopting a holistic approach in assessing academic performance.

Nevertheless, this research is not without limitations. The influence of the choice of academic ranking systems on the observed relationship introduces methodological considerations. The larger datasets of THEWUR and USWUR, compared to the smaller sample size of ARWU, highlight the importance of statistical power in detecting significant associations. This limitation suggests that the specific ranking systems chosen could impact the findings, potentially affecting the study’s outcomes. Moreover, the limited representation of universities in the QSSR Ranking (700 universities) prompts reflections of the broader generalizability of the results. Exploring additional rankings (UI Green Metric, Times Higher Education Impact Ranking) which assess universities on various sustainability aspects, could offer valuable insights into the relationship between academic performance and diverse sustainability criteria. Finally, including university-level control variables (such as faculty expertise, university budget, number of students, infrastructure) and government and policy variables into the model will ensure the robustness of the results. This necessitates further research exploration.

## Supporting information

S1 Data Data on university ranking. (XLSX)
